# Analysis and Design of Stimulus Response Curves of *E. coli*

**DOI:** 10.3390/metabo2040844

**Published:** 2012-11-12

**Authors:** Andreas Kremling, Anna Goehler, Knut Jahreis, Markus Nees, Benedikt Auerbach, Wolfgang Schmidt-Heck, Öznur Kökpinar, Robert Geffers, Ursula Rinas, Katja Bettenbrock

**Affiliations:** 1 Systems Biotechnology, Technische Universität München, Boltzmannstr. 15, Garching b. München, Germany; Email: benedikt_auerbach@web.de (B.A.); 2 University Osnabrück, Barbarastrasse 11, Osnabrück, Germany; Email: anna.goehler@gmx.de (A.G.); knut.jahreis@biologie.uni-osnabrueck.de (K.J.); 3 Max Planck Institute for Dynamics of Complex Technical Systems, Magdeburg, Germany; Email: markus.nees@mpi-magdeburg.mpg.de (M.N.); katja.bettenbrock@mpi-magdeburg.mpg.de (K.B.); 4 Hans Knoell Institute, Beutenbergstrasse 11a, Jena, Germany; Email: wolgang.schmidt-heck@hki-jena.de (W.S.-H.); 5 Helmholtz Center for Infection Research, Inhoffenstr. 7, Braunschweig, Germany; Email: koekpinar@iftc.uni-hannover.de (Ö.K.); Robert.Geffers@helmholtz-hzi.de (R.G.); Ursula.Rinas@helmholtz-hzi.de (U.R.); 6 Institute of Technical Chemistry-Life Science, Leibniz University of Hannover, Callinstr. 5, Hannover, Germany

**Keywords:** carbohydrate uptake, *Escherichia coli*, network component analysis, feedforward control, parameter estimation, gene regulatory network

## Abstract

Metabolism and signalling are tightly coupled in bacteria. Combining several theoretical approaches, a core model is presented that describes transcriptional and allosteric control of glycolysis in *Escherichia coli*. Experimental data based on microarrays, signaling components and extracellular metabolites are used to estimate kinetic parameters. A newly designed strain was used that adjusts the incoming glucose flux into the system and allows a kinetic analysis. Based on the results, prediction for intracelluar metabolite concentrations over a broad range of the growth rate could be performed and compared with data from literature.

## 1. Introduction

In bacteria, metabolism and signaling processes are tightly coupled to allow the cell to adapt efficiently to new environmental conditions. This is especially evident by the tight coupling of the metabolic and signaling functions of the bacterial phosphoenolpyruvate (PEP)-dependent phosphotransferase system (PTS). The PTS is an important uptake system, e.g., for the preferred carbon source glucose, but at the same time it represents a sensory system that signals the metabolic state of the cells. For this function, the coupling of the phosphorylation state of the different PTS proteins to the PEP to pyruvate ratio of the cell is important. A low phosphorylation state of the PTS, especially of EIIA*^Glc^*, represents a good nutritional state of the cell, while a high phosphorylation state represents hunger conditions. In addition to EIIA*^Glc^*, another protein coupled to the PTS, the FruR protein (also known as Cra), acts as a global regulator. This protein senses the concentration of fructose-1,6-bisphosphate in the cell and controls the expression of several enzymes of glycolysis and gluconeogenesis.

Central metabolism in *E. coli* is well understood from its structural properties, genetic organization and signalling characteristics, and therefore provides excellent conditions for a quantitative modelling approach. Experimental data from array experiments are available and sensor outputs as well as metabolites could also be measured. However, data is still limited to specific experimental conditions. Having a mathematical model available that is validated with experimental data from different sources (stimulus response curves, array data, dynamical experiments), it should be possible to predict the behavior for unmeasured (or hardly measurable) metabolites from model simulation studies for a large range of input conditions. Moreover, a model can help understand the architecture and allows designing new properties of the system by genetic modifications.

Glycolysis in *E. coli* can be characterized by two signalling systems where fructose-1,6-bisphosphate, PEP and pyruvate are involved as major signalling molecules. As an extension of the previous work [1,2,3,4] that did not take into account the regulation of enzyme synthesis in this pathway, we present a mathematical model that allows to describe two operating conditions: growth on carbohydrates that are taken up by a PTS, and growth on other substrates (such as lactose) taken up by other systems (named here non-PTS systems). Having a model available, the behavior of metabolite concentrations is simulated and compared with available experimental data; furthermore, new experiments that allow switching the system between different conditions were designed. In addition to previous reports, the following new aspects are included in this contribution:

- Consideration of transcriptional control of the glycolytic enzymes via transcription factor FruR and determination of the influence of the activity of FruR on gene expression via network component analysis (NCA).
- Structural analysis of the extended model. The influence of transcription factor FruR (Cra) on gene expression and metabolism is studied.
- Experimental verification of the characteristic curves for carbohydrate uptake (degree of phosphorylation of EIIA *vs*. growth rate) with a newly designed strain.
- Estimation of lumped kinetic parameters. The additional data allow an improved estimation of kinetic parameters.
- Prediction of the steady behavior of the intracellular metabolites over a broad range of the growth rate.

**Figure 1:**
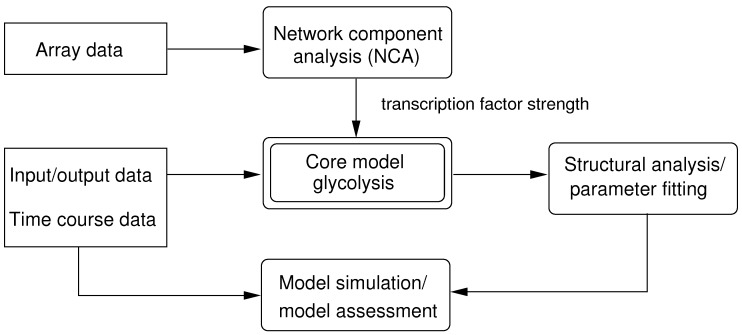
Outline on the approach. Array data, input/output data and time course data are used to set up and analyze the model. After parameter fitting, the comparison and validation of the model are performed.

The approach is summarized in Figure 1. 

### 1.1. Background

**Figure 2:**
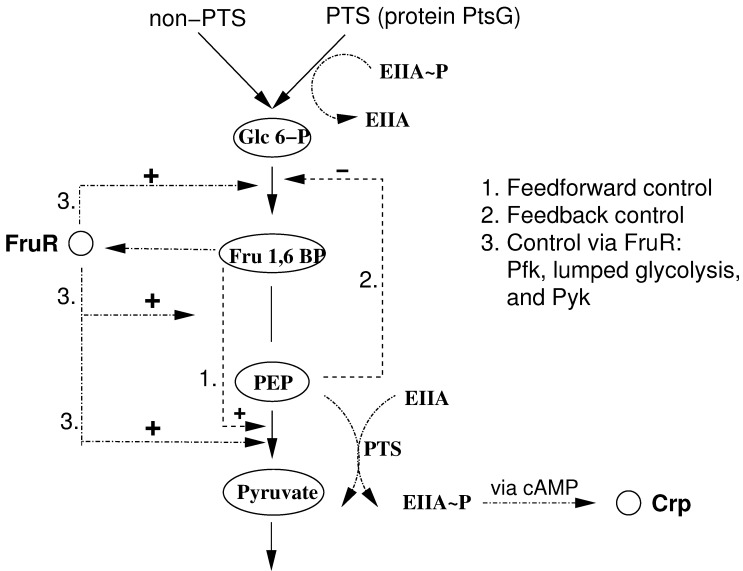
Glycolytic mode of central metabolism of *E. coli* including important regulations. Glucose is mainly taken up by PtsG, but other unspecific transport systems are also available (non-PTS). Shown are transcriptional control via FruR and allosteric control.

Figure 2 shows the core reactions of glycolysis in *E. coli*. As can be seen, the regulatory structure for growth on carbohydrates can be subdivided into genetic control via transcription factor FruR and metabolic control via feedforward and feedback loops.

Glucose represents the preferred carbohydrate of *Escherichia coli* K-12 and is taken up mainly by the glucose transporter PtsG. Several other carbohydrates feeding into the upper part of glycolysis also allow for fast growth. Organic acids such as acetate which demand an active gluconeogenesis can also be used as growth substrates but generally the growth rates on these substrates are comparatively slow. Uptake of many glycolytic substrates is catalyzed by the PTS. This system uses PEP as phosphate donor. The phosphoryl group from PEP is firstly transferred to EI in an autocatalytic reaction. EI transfers the phosphorylgroup to HPr and HPr is able to phosphorylate a number of substrate specific EIIs that catalyse uptake and phosphorylation of their respective substrates [5]. In the case of glucose the PTS represents the most important uptake system but uptake of glucose is also possible by a number of non-PTS systems such as GalP and MglABC.

Metabolism of carbohydrates is tightly controlled. Typically, the genes encoding carbohydrate uptake systems are controlled on the genetic level. In most cases induction is exerted by the specific substrate of the uptake system e.g., lactose or arabinose. In addition, many of these systems are subject to global control by cAMP∙Crp [5]. The activity of the transcription factor cAMP∙Crp is controlled on many levels. Crp concentrations in a cell can vary in response to changing growth conditions. But the most important factor determining cAMP∙Crp activity is the intracellular cAMP concentration. This is in turn determined by the phosphorylation state of the PTS protein EIIA*^Glc^*. During growth on glucose or other carbon sources allowing fast growth EIIA*^Glc^* is present mainly in its unphosphorylated form while during growth with poor carbon sources EIIA*^Glc^* is present in its phosphorylated form [1,6]. Phosphorylated EIIA*^Glc^* is able to activate adenylate cyclase thereby increasing the intracellular cAMP level and hence the amount of cAMP∙Crp.

While cAMP∙Crp controls many operons for uptake systems and peripheral metabolic enzymes as well as for enzymes of the TCA and of the respiratory chain, expression of the genes encoding enzymes of glycolysis generally is not influenced by cAMP∙Crp. Several of these genes are influenced by another PTS related regulator FruR or Cra [7]. FruR represents the repressor for the *fru* operon encoding the components of the fructose PTS as well as a 1-phosphofructokinase. In addition to its function as a specific regulator of the *fru* operon, FruR acts as an important regulator controlling or coordinating the fluxes of glycolysis and gluconeogenesis. It responds to the concentration of fructose-1-phosphate and fructose-1,6-bisphosphate in the cells [8]. Interestingly, fructose-1,6-bisphosphate is important for controlling an important point in glycolysis as it is an allosteric activator of pyruvate kinase [9], the enzyme that converts PEP to pyruvate. The same conversion is also performed by the PTS (Figure 2). Although these regulations have been characterized by different experimental approaches, a good understanding of the interplay of these regulations and of the overall effect on the fluxes in central metabolism is still lacking.

## 2. Results and Discussion

### 2.1. Structural Analysis of the Glycolysis Core Model

The model describes the steady state behavior of important metabolites of glycolysis in *E. coli*. Important components and starting points for signalling pathways are fructose-1,6-bisphosphate (ligand for transcription factor FruR), PEP and pyruvate (both determine the degree of phosphorylation of protein EIIA of the PTS). In addition, glucose 6-phosphate is taken into account as entry component into glycolysis.

The stoichiometric equations are as follows:

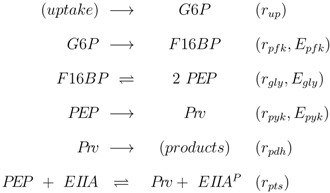
(1)
with glucose 6-phosphate *G*6*P*, phosphoenolpyruvate *PEP*, pyruvate *P_rv_*, and a lumped component of the PTS, enzyme IIA *EIIA*; *E* stands for the respective enzyme, *r* for the rate. The equations consider that a carbohydrate (PTS as well as non-PTS sugars) is fed into glycolysis via glucose-6-phosphate. The carbohydrate is metabolized by a sequence of steps with pyruvate as the final component. In reaction *r_gly_* reversible reaction steps catalyzed by the enzymes fructose bisphosphate aldolase, triose phosphate isomerase, glyceraldehyde-3-phosphate dehydrogenase, phosphoglycerate kinase, phophoglycerate mutase and enolase are lumped (*E_gly_*).

First the influence of regulation of gene expression and of allosteric control is studied. Based on the approach described in Material and Methods, the derivatives 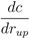 for the metabolites are calculated. Matrix *D* has the following entries:

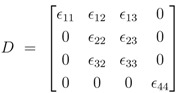
(2)
where the rows consider *r_pfk_*, *r_gly_*, *r_pyk_*, and *r_pdh_*, and the columns consider *G*6*P*, *F*16*BP*, *PEP*, and *P_rv_*. While in a previous study [4] the conditions to guarantee a negative slope for the phosphorylated form of the PTS protein EIIA were derived, the focus is now on the slope of the individual metabolites in dependence of allosteric control, genetic control, and reversibility. Table 1 summarizes all types of control and relate it to the entries in *D*. All other *ε* values, *ε*_11_ and *ε*_33_, are based on mass action and have positive values.

**Table 1:** Control schemes during growth on carbohydrates. Note that fructose-1,6-bisphosphate acts directly as allostericeffector on pyruvatekinase as well as via FruR. ^1^ Activation should be seen as double repression: fructose-1,6-bisphosphate inhibits FruR activity; FruR acts as repressor.

Explanation	
Allosteric control	
*ε*_13_ < 0	inhibition of PfkA by PEP
*ε*_32_(1) > 0	activation of Pyk by F16BP
Genetic control	
*ε*_12_ > 0	activation^1^ of PfkA by F16BP
*ε*_22_ > 0	activation^1^ of *r_gly_* by F16BP
*ε*_32_(2) > 0	activation^1^ of Pyk by F16BP
Reversibility	
*ε*_23_ < 0	reversible glycolytic reaction

The following derivatives are calculated:

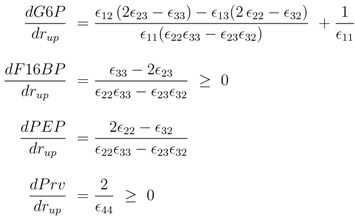
(3)
As can be seen immediately for the important metabolites fructose-1,6-bisphosphate and pyruvate, the sign is fixed and positive while the sign of glucose 6-phosphate shows a complex pattern. The sign of PEP only depends on the feedforward activation and could be positive or negative. For a more complete network of central metabolism in *E. coli*, all entries of the Jacobian matrix were determined and analyzed [10]. It turns out that most entries have fixed signs for a given flux distribution with exception of the feedforward loop represented here by *ε*_32_. Matrix *D* is related to the inverse of the Jacobian and a similar pattern can also be found here.

To further explore these equations, a more detailed analysis was done with the following kinetic approximations [4]:

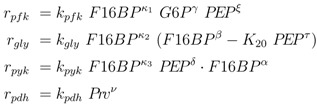
(4)
and the following kinetics for the lumped PTS system:

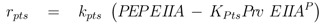
(5)


In many studies, classical saturation kinetics are chosen for the kinetic rate laws. Here, saturation is not explicitly taken into account and kinetic rate laws are approximated with power law exponents (*κ_i_* for genetic control, all other exponents for mass action and allosteric control) which are not necessarily integers. Since PEP is involved in signaling, the behavior of PEP is analyzed in more detail. As discussed in [4], PEP is a highly energetic compound and it is expected that for low growth rates this metabolite should not accumulate. However, based on the analysis of the feedforward loop [11], a monotonously decreasing behavior is necessary for a robust behavior. To resolve this conflict (a high value of the concentration of PEP is good for robustness, a low value is expected from physiological considerations), the behavior of PEP depending on the uptake rate is studied in more detail. Here, we found that a strict local maximum for PEP depending on the input flux *r_up_* could be obtained under the following conditions:

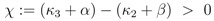
(6)

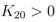
(7)
Equation (6) poses a constraint on the reaction order and the influence from transcriptional control. In order to avoid high values of PEP for small growth rates, the condition could be verified with the results of NCA and parameter estimation for the other parameters. The constraint can be interpreted as follows: the strength of control on pyruvate kinase (*κ*_3_ and *α*) should be larger than the strength of control on the lumped glycolytic reaction *r_gly_* (*κ*_2_ and *β*). The second constraint requires that the latter one is reversible. For a detailed calculation, see Appendix.

### 2.2. Influence of Transcription Factors on Gene Expression

To determine the *κ_i_* coefficients for the model, NCA was applied with three data sets. In addition, transcription factor activities could be determined as well and compared with biological knowledge on the system.

The model is similar to the previous one [3]: 32 transcriptional units are used and three transcription factors are considered (Crp, ArcA, and FruR). Although other transcription factors such as Fnr, SoxS or PdhR influence some of the genes, they are not considered explicitly here, since they are not involved in sensing metabolic fluxes in glycolysis. The number of time points is 35 (16 from growth on glucose and lactose [12], 18 from the glucose pulse experiment in this study, and 1 from growth on acetate [13]). Although strains that are used in the cited studies are different, a comparison of the growth behavior for the strains used in [12,14] reveals consistency with respect to the growth rate. Experiments in this study were performed with the same strain as in [14]. Since from [13] only one data point was taken into account, the entire data set can be regarded as consitent.

**Figure 3:**
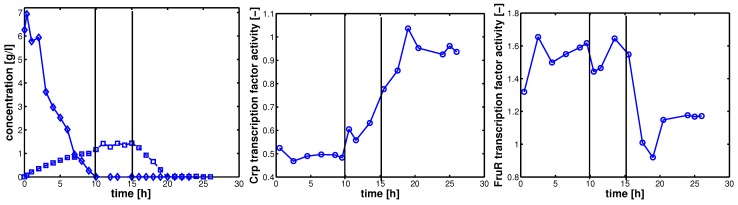
Left (plot A): experimental data for glucose (diamonds) and acetate (squares); middle (plot B) NCA results: Crp transcription factor activity; right (plot C) NCA results: FruR transcription factor activity. Circles indicate the sampling time points for array data analysis.

As described above, elements of the coupling matrix **K** and transcription factor activities **TF** are determined with NCA. Figure 3 shows the results for strain LJ110 after a glucose pulse. In a continuous culture, *E. coli* grows under glucose limited conditions. At time point zero, glucose was pulsed and the dynamics of the extracellular components and biomass was followed. Plot A shows the time course for glucose (diamonds) and acetate (squares). Three phases can be seen and are marked with vertical lines: After 10 h, glucose is depleted; at time point 15 h *E. coli* switches to growth on acetate, and after 20 h acetate is depleted. Plots B/C shows the corresponding activities of the transcription factors Crp and FruR, respectively. During growth on glucose, Crp activity is low and after depletion of glucose, Crp activity becomes more and more active. In contrast, FruR activity is high during growth on glucose (since inducer fructose-1,6-bisphosphate is expected to be high in this phase [15]), and only after shift to acetate uptake, FruR activity becomes lower as expected from other experiments [16]. 

The elements of the coupling matrix **K** that were needed for the core model of the glycolysis are summarized in Table 2. Values are given for the genes *pfkA*, *eno*, *gap*, and *pykF*. See Appendix for a complete plot with all entries of **K**.

**Table 2:** Entries *κ_i_* of the coupling matrix **K**. In further calculations the two values for *eno* and *gap* (*κ*_2_) are taken as representatives for the lumped glyoclytic reaction *r_gly_*.

gene	value	gene	value
*pfkA*	0.41 (*κ*_1_)	*eno*	0.99 (*κ*_2_)
*gap*	0.99 (*κ*_2_)	*pykF*	0.49(*κ*_3_)

A comparison of signs given in Table 2 (plus indicates activation and minus indicates repression by the transcription factor) and entries in data bases (e.g., Ecocyc [17]) shows that signs for all genes were determined correctly. In this way the influence of transcription factor FruR on gene expression of the respective enzymes in glycolysis was determined; these values were further used in the steady state and dynamic analysis of the glycolysis core model.

### 2.3. Validation with PtsG induced strains

The model presented in previous studies [2,4] was extended as described in Material and Methods and experimental data with the inducible PtsG strain were used. In this way, glucose is taken up by two systems: a non-PTS system (unspecific) and a PTS system (PtsG). With increasing amounts of IPTG, a shift from a non-PTS uptake situation to complete PTS uptake could be observed in the experimental data accompanied by increasing growth rates. Based on the data, several parameters could be determined that relate uptake by the the PTS and the non-PTS system with growth rate.

To determine the kinetic parameters a sequential approach was chosen. First, a “rough” estimation of lumped parameters via nonlinear regression analysis was performed. To do so, reversibility of the glycolytic reaction *r_gly_* and the feedback of PEP to PfkA were neglected. In this case, the degree of phosphorylation of EIIA could be described for growth on non-PTS sugars and PTS sugars in an analytical form. Moreover, as a result from our theoretical study [4], a value *v* = 1 was chosen. For non-PTS growth the degree of phosphorylation can be calculated as follows [4]:

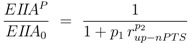
(8)
and for PTS growth:

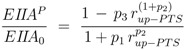
(9)
where parameters *p_i_* are lumped kinetic constants:

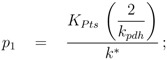
(10)

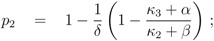
(11)

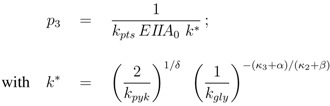
(12)


**Figure 4:**
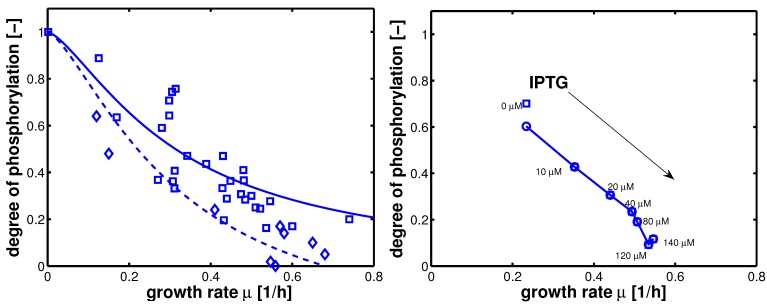
Degree of phosphorylation of EIIA (*EIIA^P^ / EIIA_0_*) versus growth rate. All data are taken from [2] (for the non-PTS data see Figure 3 left and Figure 7 left therein; for PTS data see Figure 3 right and Figure 7 right). Left (plot A): fit of the experimental data (non-PTS substrate squares, PTS substrate diamonds) with Equation system (8,9); solid lines show the simulation results for non-PTS uptake (solid) and PTS uptake (dashed). Right (plot B): For the PtsG induced strains, growth rate as well as degree of phosphorylation change (experimental data square). IPTG is increased which leads to higher expression of PtsG. With nonlinear regression the fraction of uptake via the non-PTS system is determined. See Table 3.

Considering now a simultaneous growth with both uptake systems, the uptake rate can be written as a sum:

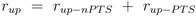
(13)
and, consequently, depending on the fraction from the overall uptake rate, the degree of phosphorylation will adjust accordingly. Given experimental data for non-PTS growth and PTS growth (data from [2]), and mixed growth (growth rates, degree of phosphorylation of EIIA for seven experiments 1–7, see Material and Methods) parameters *p_j_* as well as the fraction *f_j_* (*μ_j_*) with *j* = 1,7 of uptake via the non-PTS system could be estimated. Fraction *f_j_* (*μ_j_*) is defined as:

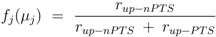
(14)
In summary, 10 parameters are estimated based on 52 data points. Results of the fit are shown in Figure 4. Table 3 summarizes the results. 

**Table 3:** Fraction *f_j_* of uptake via the non-PTS system after non-linear regression.

Experiment	1	2	3	4	5	6	7
IPTG (*μ*M)	0	10	20	40	80	120	140
*EIIA^P^ / EIIA_0_*	0.70	0.43	0.31	0.24	0.19	0.09	0.12
*f_j_*	1.00	0.83	0.64	0.50	0.35	0.00	0.15

As can be seen for experiment 1, it is estimated that glucose is completely taken up via the non-PTS uptake system; in experiment 6 a complete uptake via the PTS system through PtsG is calculated. The fraction *f_j_* could be determined perfectly in experiments 2–6 (Figure 4 right).

**Figure 5:**
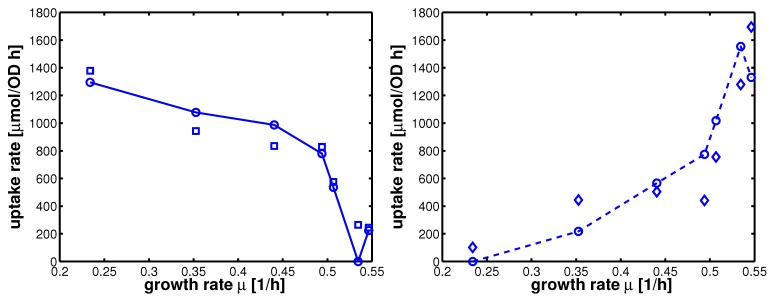
Uptake rates for non-PTS growth and PTS growth for all experiments 1-7. Left (plot A): Uptake rate of the non-PTS uptake system in dependence on the growth rate (experimental data square). Parameters of equation system (16) were estimated and simulation studies were performed (circle). Right (plot B): Uptake rate of the PTS uptake system in dependence on the growth rate (experimental data diamonds). Parameters of equation system (16) were estimated and simulation studies were performed (circle).

Having the values *f_j_*(*μ_j_*) available allows to estimate parameters of the uptake reaction kinetics for the non-PTS and the PTS uptake systems. Measurements of the degree of phosphorylation of protein EIIA were performed in the exponential growth phase. Here, glucose is abundant and it is expected that the enzymes are saturated. The non-PTS system is assumed constitutive, but based on the experiments the uptake is dependent on PtsG induction. Since no details are available for this lumped kinetic expression, an inhibition by PtsG is taken into account (black box approach). For the PTS system, a two-substrate mechanism is used as before [2]. For the two uptake systems the following kinetics are therefore chosen:

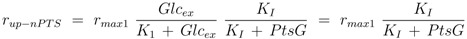
(15)

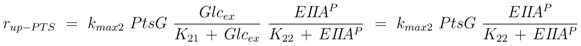
(16)
where the second equal sign is valid in case that the enzymes are saturated with glucose (*Glc_ex_* >> *K*_1,_* K*_21_). The respective uptake rates are estimated (see above) and measurements for *PtsG* and *EIIA^P^* are available. Therefore, the four unknown kinetic parameters (*r*_max1_, *k*_max2_, *K_I_*, and *K*_22_) could be estimated based on the seven experiments (Figure 5). 

Table 4 summarizes the results of the nonlinear regression of the parameters. 

**Table 4:** Kinetic parameters determined so far.

parameter	value	parameter	value
*p*_1_	1.61e-05	*p*_2_	1.43
*p*_3_	5.30e-06		
*r*_max1_	1.55e+03	*K_I_*	115.4
*k*_max2_	10.8	*K*_22_	0.034

From Equation (12) it can be seen that *p*_2_ is related to reaction order (*δ*, *α*, *β*) and the influence of FruR on the kinetic expressions (*κ*_2_, *κ*_3_). The latter two parameters are determined above via the NCA approach. From literature [9], it is known that pyruvate kinase shows a sigmoidal behavior with respect to PEP, therefore we set *δ* = 2. Rearranging Equation (12) and with results from above leads to:

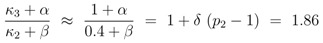
(17)
Enzymes in the glycolysis are described with a hyperbolic behavior [18] and we set *β* = 1. As a result, the influence of the feedforward activation by fructose-1,6-bisphosphate can be calculated to *α* = 1.53 . Taking into account that the sigmoidal behavior of the pyruvate kinase was described with *δ* = 2 that corresponds to the number of domains of the system, the value for *α* is in good agreement since it should also reflect the number of the domains (actually pyruvate kinase is a tetramer; however, the chosen Hill coefficients are only approximations and the simplest value was chosen; for *δ* = 4, *α* ≈ 3 is calculated).

**Figure 6:**
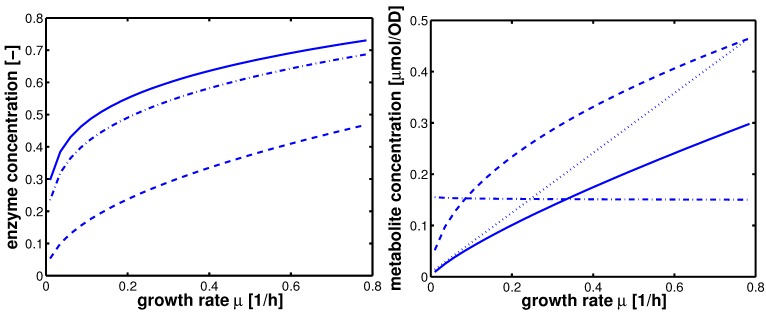
Left (plot A): Course of the enzymes PfkA (solid), lumped glycolysis (dashed), and Pyk (dash-dotted) with the growth rate. Right (plot B): Course of the metabolites glucose-6-phosphate (solid), fructose-1,6-bisphosphate (dashed), PEP (dash-dotted), and pyruvate (dotted) with the growth rate.

From Equations (10) and (12) the following estimation could be done:

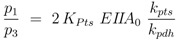
(18)
Taking values for *K_Pts_* and *EIIA_0_* from literature [2], the ratio between the PTS system constant and the pyruvate dehydrogenase constant could be calculated: 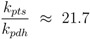. The values show a high capacity of the PTS chain in comparison with glycolytic fluxes. It is expected that sudden changes in the uptake rate should be seen immediately also in the degree of phosphorylation. This was demonstrated experimentally [14]. The course of the metabolites and the enzymes are shown in Figure 6. Based on the model structure and the estimated parameters, all enzymes and metabolites show an increasing behavior while PEP is nearly constant over the growth rate.

### 2.4. Dynamic Model

**Figure 7:**
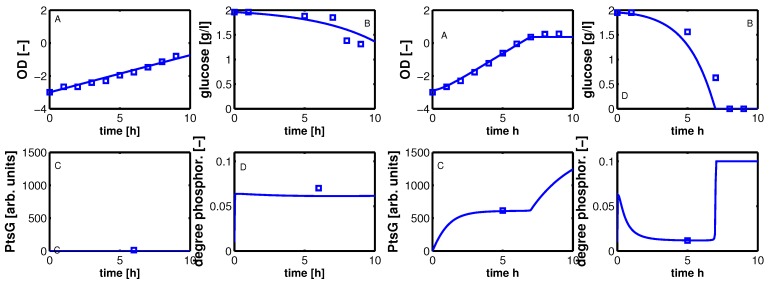
Dynamic model simulation and comparison with experimental data. A: optical density, B: glucose in g/l, C: PtsG in arbitrary units, D: degree of phosphorylated EIIA (dimensionless). Left: IPTG 0 *μ*M; Right IPTG 140 *μ*M.

Having the model parameters for the uptake systems available, the complete system including PtsG induction kinetics (see Appendix) was simulated with fine-tuned parameters (empirical tuning of the parameters). First, time course data with simulated and experimental data are shown in Figure 7 for selected values of IPTG.

Shown are data for optical density (plot A), extracellular glucose concentration (plot B), PtsG (plot C), and EIIA phosphorylated (plot D) for no IPTG (left) and maximal IPTG concentration (right). The calculation of the optical density in plot A shows a very good agreement with the experimental data; glucose uptake for high concentrations of IPTG could not be reproduced accurately as the yield coefficients for each experiment were different and in the calculations, a mean value was used. Furthermore, the fit of the parameters for glucose uptake (Figure 5) also shows differences between simulated and experimental values that can be seen here again. For a high value of IPTG, the value of PtsG measured in the exponential phase could be described as well as the degree of phosphorylation of EIIA.

**Figure 8:**
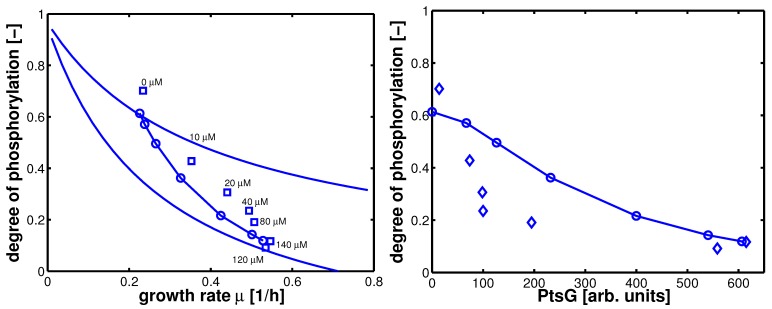
Left (plot A): Degree of phosphorylation of EIIA versus growth rate. Shown are simulation results for growth on non-PTS carbohydrates (upper solid line) and for growth on PTS carbohydrates (lower solid line). Values for the experiments 1–7 are taken in the exponential phase (squares). Simulation results for experiments 1–7 are shown with symbols (circles). Right (plot B): Degree of phosphorylation of EIIA versus level of PtsG. Values for the experiments 1–7 are taken in the exponential phase (diamonds). Simulation results for experiments 1–7 are shown with symbols (circles).

Steady state values (taken in the exponential phase) are considered and compared with simulation results (Figure 8). Plot A shows simulation results for growth on non-PTS (upper curve) and PTS (lower curve) carbohydrates. For small uptake rates, both curves converge. For large growth rates, the PTS reaches its capacity limit since the energy for the transport is generated in the glyoclysis itself.

For increasing values of IPTG, the system moves from one curve to the other, indicating a change of the uptake system based on the induction of PtsG: the degree of phosphorylation decreases while the growth rate increases. A comparison of the experimental data with the simulation data reveals differences in the growth rate. The first (experiment 1) and last (experiment 7) data point are fitted with high accuracy while in the other experiments larger deviations could be seen. As indicated before, the main reason for the deviations is most probably the variation in the yield biomass/glucose and the choice of simple rate laws. Table 5 summarizes the simulated and the experimental data for growth rate and the two uptake rates. Plot B in Figure 8 shows the relationship between PtsG and the degree of phosphorylation of EIIA.

**Table 5:** Summary of the simulation results. Comparison between measured quantities and simulated quantities for experiments 1–7. First column: growth rate *μ*. *μ* is given in 1/h. Second and third columns: uptake rates via non-PTS system and PTS system. The uptake rates are given in 10^3^ *μ*mol/ OD units h.

IPTG		growth rate		non-PTS uptake		PTS uptake	
		sim	ex	sim	ex	sim	ex
1.	0	0.23	0.23	1.76	1.67	0.00	0.00
2.	10	0.24	0.35	1.35	2.10	0.50	0.42
3.	20	0.27	0.44	1.12	2.00	0.94	1.15
4.	40	0.33	0.49	0.85	1.77	1.69	1.76
5.	80	0.43	0.51	0.56	1.25	2.75	2.37
6.	120	0.50	0.54	0.43	0.00	3.47	3.82
7.	140	0.53	0.55	0.36	0.56	3.74	3.34

### 2.5. Discussion

Mathematical modeling can be a powerful tool to analyze systems that are hardly observable. Here, we use a simple core model for glycolysis of *E. coli* to predict semi-quantitatively the steady state behavior for central metabolites in dependence on the growth rate (for downloading all files and comments see information given in the Appendix). Glycolysis is an important reaction system since some of the metabolites such as fructose-1,6-bisphosphate, PEP and pyruvate are closely related to signalling units that trigger the important transcription factors FruR and Crp. While experimental data for metabolite concentrations [19] and mathematical models [18] are available for specific situations—normally covering one single growth rate—complete data sets for a broad range of growth rates are scarce. More complete models for central metabolsim were presented [20,21], however, a quantitative comparison with experimental data is missing. Therefore, these models are not suited for a fair comparison. In [22] a detailed mathematical model similar to a model published by us [14] was presented but failed to predict genetic modifications. A comparison of modeling approaches and a presentation of the current “state of the art” on this topic can be found in [23]. To summarize, mathematical models to describe carbohydrate uptake and metabolism are available, but fail in reproducing experimental data or fail in predicting new experiments.

In previous studies, we already analyzed the input/output relationship to describe a characteristic curve that relates growth rate for a number of carbohydrates and the degree of phosphorylation of EIIA, an important metabolite of the PTS. Other groups focus on structural properties of the same system [10] or on the relationship between control, metabolites and fluxes through the system [20]. In this study, new experimental data is presented to extend our current model by taking into account the transcription factor activities, and experiments that are designed to modify the already available input/output characteristic curves in such a way that kinetic parameters can be estimated with higher accuracy are performed. Here, a strain is used that allows adjusting the level of the main glucose uptake system, namely PtsG, with IPTG as inducer. In this way, different amounts of the PtsG uptake system could be adjusted and data could be used to estimate parameters related to metabolites that could not be measured. In contrast to previous publications the parameter fitting procedure was modified: first the lumped parameters were estimated via nonlinear regression, finally all parameters were adjusted based on the estimation and literature data. 

Results from NCA allow to determine the influence of transcription factor activities on a set of selected genes. Data that were used in a different study were complemented with new experiments. In this experiment, glucose was pulsed to a culture growing under glucose limitation. Glucose was immediately taken up and after 10 h glucose was depleted. Acetate is produced during growth on glucose and consumed after 15 h. The different energy sources lead to different transcription factor activities that could be estimated with NCA. Furthermore, the influence of each transcription factor on each gene is described with a coupling factor *κ*. A crucial issue is the verification of the elements of the coupling matrix. In most studies—also in the first publication that introduces the method—the signs of the entries were not validated with entries of databases. In our previous study [3] we already could show that an agreement for all entries is hardly possible but shows 70%–100% correct values. In the current study the error for transcription factor FruR is around 10%, that is, only one sign, here for the *icd* gene (isocitrate dehydrogenase in the TCA) is different from the data base entry. The values for *pfkA*, *eno*, *gap*, and *pyk* are determined from the experiments and are taken into account in further parts of the study. Interestingly the values for *eno* and *gap* are similar and are integrated into a single value for the lumped glycolytic reaction *r_gly_*.

A structural analysis of the core model including all regulatory features was performed to calculate the behaviour of the intracellular metabolites of the core model (glucose-6-phosphate, fructose-1,6-bisphosphate, PEP and pyruvate). While the signs for fructose-1,6-bisphosphate and pyruvate are fixed and show positive values, it is expected that both metabolites show increasing values if the uptake rate is increasing. In contrast, the signs of PEP and glucose-6-phosphate are not fixed. Since PEP is an important metabolite for the PTS and the PEP/pyruvate ratio determines the degree of phosphorylation, the behavior of PEP in dependence on the growth rate was further explored. In a previous study, we analyzed the robustness of a simplified version of the model and it turns out that a monotonous decreasing course of PEP is more favourable with respect to robustness [11]. In this study, conditions for the extended model were derived allowing the course of PEP over the growth rate to show a maximum. These constraints are related to the regulatory properties on the transcriptional level (*κ*_2_ and *κ*_3_) and kinetic properties (*α*, *β*, *K*_20_).

**Figure 9:**
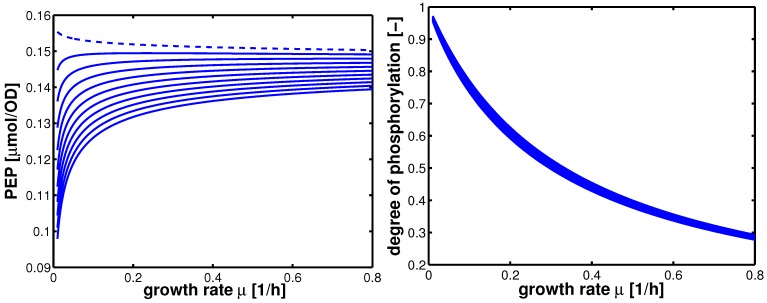
Left (plot A): course of PEP for different values of *K*_20_ (dashed curve: *K*_20_ = 0). Right (plot B): course of phosphorylated EIIA for different values of *K*_20_. *K*_20_ was varied between 0.05 and 0.5.

Based on the first “rough” estimation of the model parameters, the influence of the reversibility of the glycolytic reaction *r_gly_* was studied with numerical simulations. Parameter estimation reveals that condition (6) was fulfilled. Doing so, the course of PEP depends on the value of *K*_20_ (equilibrium constant of the reversible glycolytic reaction *r_gly_*). Figure 9 shows the course of PEP for different values of *K*_20_ (plot A) and the course of phosphorylated EIIA. As can be seen in Figure 9, an extremum is reached for a small value of *K*_20_ in the range of the growth rate considered here. In all other cases, PEP is monotonously increasing. The course of phosphorylated EIIA shows a very low sensitivity with respect to *K*_20_ (plot B). The same is true for fructose-1,6-bisphosphate (data not shown).

Experimental data to verify the simulations are found. Several studies focus on single growth conditions or on specific stimulations of the system [18,19]. In [24] *E. coli* was starved for carbohydrates and nitrogen. This situation reflects a move from a high growth rate to a very low growth rate. Figure 4 in [24] shows time course data for PEP and fructose-1,6-bisphosphate for carbon starvation. After stimulation, PEP increases very fast up to a factor of 64 but then decreases, and after 8 hours the former steady state is almost reached; in contrast fructose-1,6-bisphosphate decreases fast and remains at the new steady state during the remaining time of the experiment. In [25] several stimulations were performed and PEP was measured. For a classical experiment when *E. coli* is growing on glucose and lactose, the dynamic of PEP could also be monitored. For both growth phases, the level of PEP is nearly constant. In an A-stat experiment different metabolites of central metabolism were monitored [15]. Although the data are noisy, the level of fructose-1,6-bisphosphate and glyceraldehyde-3-phosphate show a monotone increasing correlation with the growth rate. To summarize, there is experimental evidence that the simulation results predicted using this model (see Figure 6) reflect the true intracellular behavior.

With a newly designed strain that allows to adjust the level of PtsG, it was possible to “move” from one branch of the characteristic curve of phosphorylated EIIA (non-PTS case) to the other branch (PTS case). The experiments are designed such that glucose is taken up by the PTS and also by non-specific uptake systems. With the data of these experiments, it was possible to approach different points on the uptake kinetics for parameter estimation. While the data for the degree of phosphorylation were taken from previous experiments, the data with the new strain confirmed the relationship already published.

The approach shows that an understanding of the intracellular network is required to design and modify such network and offers new possibilities in medical and biotechnological applications. 

## 3. Material and Methods

### 3.1. Experimental Data

#### 3.1.1. Strain Construction

A deletion of the *galP* gene was transduced from the strain JC7623Δ(*galP::kan*) into the genomes of the strains LJ121 (LJ110Δ(*ptsG::cat*) man-8 zea-225::Tn10) and LJ130 (LJ110 Δ(*manXYZ::cat*)) [26] to generate the strains JGA1 and JGA2, respectively. Next, the chromosomal markers of the new strains were confirmed. These strains were generated as a test strain incapable of glucose uptake (JGA1) and a control strain (JGA2) that only provides the chromosomal *ptsG* transporter gene for glucose uptake. The wild type genes encoding the galactose ABC transporter MglBAC, which is able to transport glucose with very low affinity, are present in all strains. The plasmids pRR48 or pRRGH (pRR48 with the *ptsG* gene under the control of a *tac* promoter [27]) were transformed into the JGA1 and JGA2 strains.

The growth behavior of the strains JGA1/pRR48 (no *ptsG* expression), JGA1/pRRGH (basal expression level of *ptsG* encoded on the plasmid), and JGA2/pRR48 (chromosomal *ptsG* expression level) were monitored in minimal medium with ampicillin and either glycerol or glucose as a carbon source. Utilizing glycerol as carbon source, the strains showed similar generation times (JGA1/pRR48: *μ* = 0.26 h^−1^; JGA1/pRRGH: *μ* = 0.27 h^−1^; JGA2/pRR48 *μ* = 0.28 h^−1^). Whereas the growth rates in minimal medium supplied with glucose revealed the expected differences due to different *ptsG* genotypes (JGA1/pRR48: *μ* = 0.04 h^−1^; JGA1/pRRGH: *μ* = 0.19 h^−1^; JGA2/pRR48 *μ* = 0.30 h^−1^) the addition of IPTG to the medium resulted in an induction of the *tac* promoter in front of the encoded *ptsG* gene and hence to an increase in available EIICB*^Glc^* protein. This was again correlated with an enhanced rate of glucose uptake and utilization, resulting in increased growth rates (seven experiments were performed with 0, 10, 20, 40, 80, 120, and 140 *μ*M IPTG).

The amount of EIICB*^Glc^* within such a culture was directly compared with the amount of glucose transporter protein in the strain JKA4 when grown in minimal medium with glucose. The plasmid encoded *ptsG* gene on pRRGH is fused to a His-tag encoding sequence and the latter strain carries the chromosomally encoded and hence physiologically regulated *ptsG* gene, also fused with a His-tag encoding sequence. Western blot analysis was performed with specific penta-his antibodies and the signals quantified, detecting equivalent amounts of EIICB*^Glc^* for the two strains.

For determining the degree of phosphorylation, cells were harvested from cultures growing with various induction conditions as described above and tested in a second Western blot analysis. In this case, the sample preparation was carried out according to a protocol that is suitable for immediately freezing the phosphorylation status of proteins in the sample. The final step involved the quantification of the Western blot signals using the LICOR Odyssey software. In each approach and Western blot the amount of EIICB*^Glc^* from the culture supplemented with 50 *μ*M IPTG was set to 100% and the strength of signals detected in cultures supplemented with less IPTG were referenced to this value. The degree of phosphorylation of EIIA*^Glc^* was calculated using the ratio of the phosphorylated EIIA signal (upper band) to the entire signal of EIIA (upper and lower signal) for each sample.

#### 3.1.2. Microarrays

Array data for NCA were compiled from a glucose pulse experiment. Cells of *E. coli* LJ110 were grown in a 1 L chemostat culture at 28°C with 8 g/L glucose at a dilution rate of 0.072 1/h. Medium composition was essentially as described in [28]. O^2^ and CO^2^ concentrations in the off gas were monitored continuously. After the culture displayed steady state growth as displayed by stable biomass and stable CO^2^ production, glucose was pulsed to 10 g/L. Immediately before and at defined time points after the pulse samples were taken for microarray analysis as well as for the determination of extracellular metabolites.

Extracellular metabolites glucose, acetate, formate and lactate were measured by using the respective enzymatic test kits of r-biopharm according to the instructions provided by the manufacturer but scaled for the use of 96 well plates. Microarray analysis was performed on microarrays purchased from Agilent.

Total RNA was isolated from the cells using the protocol accompanying the RNeasy Mini Kit (Qiagen; Hilden, Germany). Quality and integrity of the total RNA was controlled on an Agilent Technologies 2100 Bioanalyzer (Agilent Technologies; Waldbronn, Germany). 200 ng of total RNA were applied for Cy3-labelling reaction using the MessageAmp II-Bacteria Kit according to supplier’s recommendation (Ambion; Kaufungen, Germany). As a result of IVT (*in vitro* transcription) reaction using aminoallyl-dUTP antisense aRNA were generated and subsequently coupled with fluorescent dye Cy3. Cy3-labeled aRNA was hybridized to Agilent’s 8 × 15k *E. coli* microarray (Agilent Technologies; Waldbronn, Germany, AMADID 020097) for 16 h at 68°C and scanned using the Agilent DNA Microarray Scanner. Expression values (raw data) were calculated by the software package Feature Extraction 10.5.1.1 (Agilent Technologies; Waldbronn, Germany) using default values for GE1_105_Dec08 extraction protocol.

### 3.2. Mathematical Model

#### 3.2.1. Network Component Analysis

To determine the influence of the transcription factors Crp, ArcA and FruR on the genes of central metabolism, Network Component Analysis (NCA) [29] was applied to several data sets:

- diauxic growth on glucose and lactose [12]
- glucose pulse experiment (this study)
- growth on acetate [13]

NCA allows a semi-quantitative description of gene expression based on measured transcriptomic data. In brief, the approach is as follows: The number of selected genes is *N* and the number of selected transcription factors is *m*. The dynamics of a single gene (*i*) is described with an ordinary differential equation:

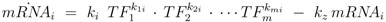
(19)
with the last term describing the degradation of the mRNA. Parameters *k_ji_* are related to the strength of each transcription factor *TF_j_* binding to the respective control sequence: if *k_ji_* > 0 then the transcription factor is an activator, while *k_ji_* < 0 points to an inhibition. Assuming that the dynamics of mRNA is faster than protein synthesis, a steady-state assumption holds true and the following equation results after fixing a set point (subscript 0):

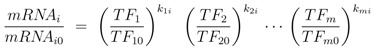
(20)
Taking logarithm (log_2_) leads to:

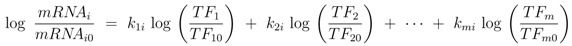
(21)
which can be written in matrix form:

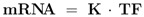
(22)
with **K** is *N* × *m* coupling matrix representing the effect of each transcription factor on the respective gene, and **TF** is an * m* × *t_k_* matrix of transcription factor activities (*t_k_* is again the number of available data points). The aim is now to decompose matrix **mRNA** to get both **K** as well as **TF**. Note that the entries of **K** have to be specified before (value 0 if a transcription factor is not involved in the regulation of the gene and 1 as starting value for the algorithm, if a transcription factor is involved) the algorithm starts, that is, the structure of the model has to be given and NCA determines the coupling strength and the time course of transcription factor activities. To solve the problem, the following objective function is minimised:

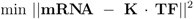
(23)
considering the difference between measured data and model simulation. Further details and the algorithm as MATLAB file can be found in the original paper [29].

The data set considered in this study comprises 50 transcriptional units (75 genes) and *m* = 3 transcription factors (Crp, ArcA, and FruR). After filtering out genes with no entry in the database (no experimental evidence that the gene is under control of one of the transcription factors) the final model contains *N* = 33 genes, representing the central metabolism. The choice is based on prerequisites of the algorithm and the experimental conditions chosen. Therefore, transcription factor Fnr related to genes that are involved in oxygen consumption is not considered. Also, several other transcription factors cannot be integrated or are not significant, e.g., considering transcription factor Fis showed that this transcription factor has only marginal influence on the calculations.

### 3.2.2. Steady State Network Analysis

According to a previous study the metabolic network of the form

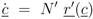
(24)
is considered with the vector of internal concentrations *c*, the non-negative rate vector *r'*(*c*) of external and internal rates and a fixed stoichiometric matrix *N’* [4]. The rate vector *r'* will be partitioned into an unknown rate vector *r* of internal rates and into a known rate vector *r_up_* of free input fluxes, here, uptake rate and known rates for biosynthesis. The stoichiometric matrix *N’* will be partitioned accordingly into sub-matrices *N* and *N_up_*. The admissible steady states of the network are then given by the solutions *r* of

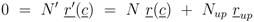
(25)
It is assumed that the submatrix *N* of the stoichiometric matrix *N’* to be an invertible square matrix so that

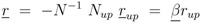
(26)
is the unique solution of Equation (25), in case that only a single uptake rate is considered.

The influence of the input *r_up_* on the concentrations *c*(*r_up_*) in steady-state might be calculated from

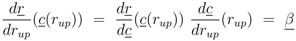
(27)
where 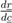 denotes the Jacobian matrix (or elasticities) of the rates *r_i_* with respect to the concentrations *c_j_*:

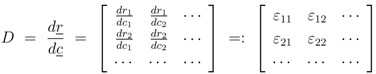
(28)
In case the Jacobian *D*(*c*(*r_up_*)) is invertible, the dependency of the steady-state concentration *c*(*r_up_*) on the input *r_up_* is uniquely determined by

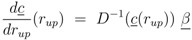
(29)
Equation (29) allows to calculate the slope for each component in dependence of the elasticities given in *D*. This will be important for the characterization of the steady-state solutions for given uptake rates.

#### 3.2.3. Dynamic Network Analysis

Based on the reaction scheme above, differential equations (o.d.e.) are set up and kinetic parameters are either taken from network component analysis or estimated based on the experimental data. The dynamic system comprises differential equations for substrates (glucose, acetate), biomass, metabolites glucose 6-phosphate, fructose 1,6-bisphosphate, PEP and pyruvate. Furthermore, gene expression and control via FruR of the following enzymes is considered: phosphofruktokinase (PfkA), pyruvate kinase (pykF), and a lumped enzyme for glycolytic reactions. Simulation studies and parameter estimation are performed with MATLAB.

Some of the enzymes of glycolysis are subject to transcriptional control by FruR. These enzymes are taken into account in the model with additional equations. Taking the simplified form for the enzymes according to [3], the steady state value of an enzyme is proportional to the transcription factor activity that in turn is determined by the concentration of the metabolite fructose-1,6-bisphosphate (*F*16*BP*). Therefore, the steady state of the enzyme corresponds directly to ligand concentration and the respective parameter *κ_i_* is determined with NCA (see Material and Methods):

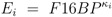
(30)
with *κ_i_* being the entry in the coupling matrix **K**.

The complete dynamical system reads as follows:

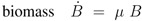
(31)

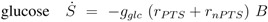
(32)

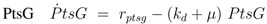
(33)

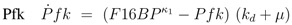
(34)

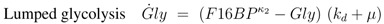
(35)

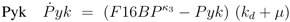
(36)

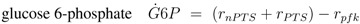
(37)

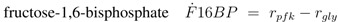
(38)

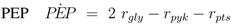
(39)

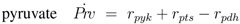
(40)

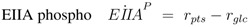
(41)


In addition to the kinetic expressions given in the text, the following rate law is used to calculate the growth rate *μ* based on the yield coefficient *Y*:

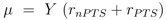
(42)
Yield coefficient *Y* was determined as follows: for the seven experiments, the individual yield coefficients were determined by linear regression, finally a mean value for all experiments was calculated. The following kinetic parameters in Table 6 are used for dynamical simulation studies:

**Table 6:** Summary of the kinetic parameters. *g_glc_* molecular weight for glucose. Basic units are OD (for biomass), *μ*mol (for substrate), and hours (for time). *^a^* Taking a value of 0.32 g/OD (determined experimentally for a different study) this corresponds to a yield of 0.23 g dry weight per g glucose; *^b^* Parameter is fine-tuned; *^c^* feedback is not considered in the study since only glucose-6-phosphate is affected.

*Y*	1.29 10^−4^ OD/*μ*mol*^a^*	*g_glc_*	180.2 10^−3^ g/*μ*mol
*k_d_*	0.3 1/h		
*κ*_1_	0.41	*κ*_2_	1.0
*κ*_3_	0.49		
*k_pfk_*	2.80 10^4^ *μ*mol /OD h	*k_gly_*	2.80 10^4^ *μ*mol /OD h
*k_pyk_*	2.53 10^6^ *μ*mol /OD h	*k_pdh_*	2.40 10^4^ *μ*mol /OD h
*k_pts_*	52 10^4^ *μ*mol /OD h	*K_pts_*	0.7
*EIIA*_0_	0.1 *μ*mol /OD		
*r*_max1_	1.55 10^3^ *μ*mol /OD h	*K_I_*	115.4
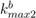	8.0 *μ*mol /OD [arb. units] h	*K*_22_	0.034
*γ*	1.0	*ξ^c^*	0

## Figures and Tables

**Figure 10:**
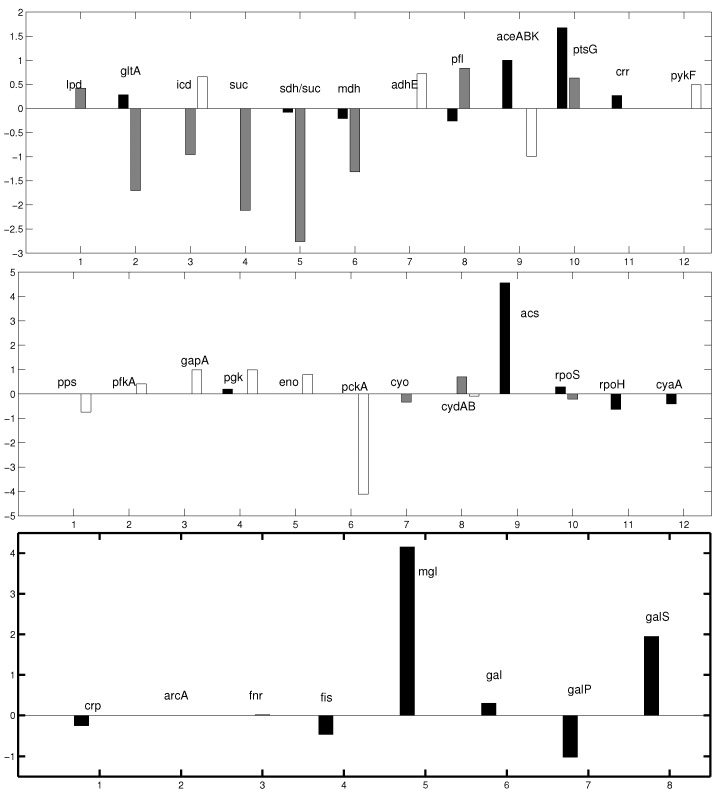
Entries of matrix *K*. Top: Genes 1–12, middle: genes 13–24, bottom: genes 25–32. Names of the genes are given in the plot. Colors indicate transcription factors Crp (black), ArcA (gray), and FruR (white).

**Figure 11:**
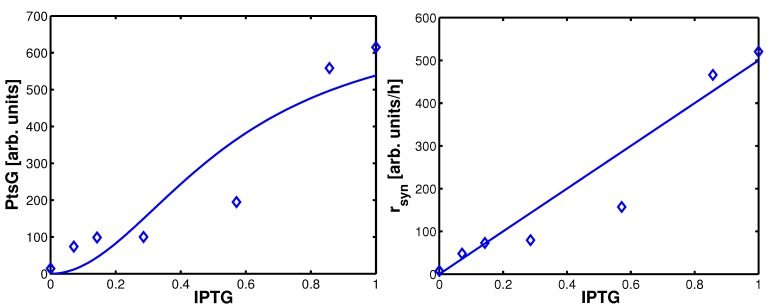
Left (plot A): Induction kinetics for PtsG for increasing concentrations of IPTG (1 ≡ 140 *μ*M). Right (plot B): Rate of synthesis in dependence on IPTG (1 ≡ 140 *μ*M). The relationship between IPTG and the rate of synthesis is almost linear with a slope of ≈ 500 arb. units/ *μ*M h.

## Appendix

### Strict Local Maximum for PEP

The two constraints mentioned in the main text:

(43)

can be justified as follows: For *K*_20_ = 0 the steady state solution for *PEP*(*u*) can be computed algebraic from differential equations (in the following *u* is used instead *r_up_*):

(44)

(45)

(46)

(47)

Thus in this case *PEP*(*u*) is monotonously increasing for *χ* < 0, constant for *χ* = 0 and monotonously decreasing for *χ* > 0. Overall when *K*_20_ = 0 the course of *PEP*(*u*) is monotonous.

In the case *K*_20_ > 0, which is equal to a reversible reaction *r_gly_*, the steady state solution of *PFP*(*u*) cannot be calculated algebraically, but its derivative  can be computed:

(48)

For positive metabolite concentrations *F* and *PEP*, which holds for *u* > 0, the expression

(49)

is positive. Therefore the existence of a strict local maximum in the run of *PFP*(*u*) is equivalent to a change of the sign of the expression

(50)

from positive to negative. Equation (50) is given by

(51)

Hence the sign of expression (50) equals the sign of

(52)

where  and  are positive constants.

When *K*_20_ > 0 one receives *F*(0) = 0 = *PEP*(0). This holds true since by eliminating *PFP*(*u*) from the differential equations, *F*(*u*) can be computed as the root of the expression

(53)

A solution to this expression exists for all *u* ≥ 0 since *K*_20_ > 0 and . Furthermore for *u* = 0 the unique solution of Equation (53) is given by *F*(0) = 0, which implies *PEP*(0) = 0 since from 0 = *u* − *r_gly_* follows

(54)

Since *F*(*u*) and *PEP*(*u*) are continuous  for very small values of *u* (note that this does not hold for *K*_20_ = 0 and *χ* > 0, since in this case  for all *u* > 0 according to Equation (47)). Thus in the case *K*_20_ > 0, independent of the value of *χ*, the function *PEP*(*u*) is at first increasing, and therefore expression (52) is at first positive. The behaviour of *F*(*u*) for *u* → ∞ can be derived from Equation (53) as well: in this case also *F*(*u*) → ∞ is mandatory to fulfill Equation (53).

Since  for *χ* > 0 and since *PEP* is not decreasing while *f* (*u*) ≤ C, the function *f*(*u*) is at first monotonously increasing and there even has to exist a *û* > 0 such that *f* (*û*) > C. Therefore the sign of expression (52) changes from positive to negative, which equals the existence of a strict local maximum in the course of *PEP*. In the case *χ* ≤ 0 the expressions *F*(*u*)*^χ^* and *F*(*u*)^−^*^β^* are non-increasing. Furthermore when *f* (*u*) = C also *PEP*(*u*) stops to increase. Therefore the function *f*(*u*) is bounded by *C*, and hence expression (52) is always positive, which shows that in this case one obtains a monotonously increasing function *PFP*(*u*). Overall, there is a strict local maximum in the course of *PEP*(*u*), while *K*_20_ > 0, is equivalent to (*κ*_3_ + *α*) − (*κ*_2_ + *β*) =: *χ* > 0.

### NCA Results

NCA provides all entries *κ_i_* for all genes and all transcription factors. In the model Crp, ArcA and FruR were used as transcription factors and 32 transcriptional units were analyzed. Figure 10 shows all values for matrix *K*.

### Kinetics of PtsG Induction

Experimental data with an inducible PtsG strain (see Material and Methods) were generated by different additions of IPTG. With IPTG the amount of PtsG protein in the cell could be adjusted. The steady state data (7 data points) allow to determine kinetic parameters for enzyme synthesis. During steady state, the rate of synthesis can be calculated according to the following equation:

(55)

Since both the growth rate *μ* and PtsG data are available the rate *r_syn_* can be estimated. Figure 11 shows the induction kinetics as well as the rate of synthesis of PtsG.

### Software to Follow the Results

All calculations were performed with MATLAB. Files are stored at http://sourceforge.net/projects/sysbioecolimode/ and can be downloaded.
